# Inactivation of the *MSTN* gene expression changes the composition and function of the gut microbiome in sheep

**DOI:** 10.1186/s12866-022-02687-8

**Published:** 2022-11-11

**Authors:** Chenchen Du, Xianhui Zhou, Ke Zhang, Shuhong Huang, Xiaolong Wang, Shiwei Zhou, Yulin Chen

**Affiliations:** College of Animal Science and Technology, Northwest A&F University, Yangling, 712100 China

**Keywords:** Myostatin edited, Gut microbiome, Microbiota function, Sheep

## Abstract

**Background:**

Myostatin (*MSTN*) negatively regulates the muscle growth in animals and *MSTN* deficient sheep have been widely reported previously. The goal of this study was to explore how *MSTN* inactivation influences their gut microbiota composition and potential functions.

**Results:**

We compared the slaughter parameters and meat quality of 3 *MSTN*-edited male sheep and 3 wild-type male sheep, and analyzed the gut microbiome of the *MSTN*-edited sheep (8 female and 8 male sheep) and wild-type sheep (8 female and 8 male sheep) through metagenomic sequencing. The results showed that the body weight, carcass weight and eye muscle area of *MSTN*-edited sheep were significantly higher, but there were no significant differences in the meat quality indexes. At the microbial level, the alpha diversity was significantly higher in the *MSTN*-edited sheep (*P* < 0.05), and the microbial composition was significantly different by PCoA analysis in the *MSTN*-edited and wild-type sheep. The abundance of Firmicutes significantly increased and Bacteroidota significantly decreased in the *MSTN*-edited sheep. At genus level, the abundance of *Flavonifractor, Subdoligranulum, Ruthenibacterium, Agathobaculum, Anaerotignum, Oribacterium* and *Lactobacillus* were significantly increased in the *MSTN*-edited sheep (*P* < 0.05). Further analysis of functional differences was found that the carotenoid biosynthesis was significantly increased and the peroxisome, apoptosis, ferroptosis, N-glycan biosynthesis, thermogenesis, and adipocytokines pathways were decreased in the *MSTN*-edited sheep (*P* < 0.05). Moreover, carbohydrate-active enzymes (CAZymes) results certified the abundance of the GH13_39, GH4, GH137, GH71 and PL17 were upregulated, and the GT41 and CBM20 were downregulated in the *MSTN*-edited sheep (*P* < 0.05).

**Conclusions:**

Our study suggested that *MSTN* inactivation remarkably influenced the composition and potential function of hindgut microbial communities of the sheep, and significantly promoted growth performance without affecting meat quality.

**Supplementary Information:**

The online version contains supplementary material available at 10.1186/s12866-022-02687-8.

## Background

Myostatin (*MSTN*), also known as growth differentiation factor 8 (*GDF-8*), is highly expressed in skeletal muscle tissue and negatively regulates the development of muscles [1]. Inactivation of *MSTN* expression causes muscle hyperplasia and hypertrophy, which has been observed in mice [1], cattle [2–4], dogs [5, 6], sheep [7], goats [8, 9], pigs [10, 11] and even humans [12]. In view of the great value of *MSTN* in animal breeding, we produced the *MSTN*-edited Tan sheep by CRISPR/Cas9, and found that editing of the *MSTN* significantly increased diameter of myofibers, the average daily gain (ADG) and body weight in sheep [13, 14].

Host-gut microbiome interaction has attracted significant research interest. Many studies illustrate that host genetic factors strongly affect the composition of gut microbiota. For instance, mice models revealed overexpression of the *DEFA5* gene significantly decreased the relative intestinal abundance of Firmicutes but increased that of Bacteroidota [15]. In related research, colitis mice model confirmed that inhibiting the expression of *CARD9* disrupted the diversity and abundance of the gut microbiome [16]. Activating the expression of the *GUCY2C* gene causes dysbiosis in the gut lumen, decreases the diversity of the fecal microbiota and the abundance of probiotics and increases the abundance of pathogenic microorganisms [17]. In addition, the gut microbiome plays a crucial role in shaping host phenotypes. For example, mice models verified the introduction of *Lactobacillus plantarum* in the germ-free mice buffered their chronic undernutrition [18]. A related study revealed oral administration of fecal samples from healthy calves increased the abundance of *Porphyromonadaceae*, relieved diarrhea, and improved the overall growth of those with diarrhea [19]. These findings suggest that the gut microbiome has a synergistic effect on host growth and health. Previous studies have revealed a positive change of microbiota in pigs and cattle following mutation of the *MSTN* [20, 21]. However, the effect of *MSTN* gene editing on the composition and function of gut microbiota in sheep is lacking.

In the present study, we used metagenomic sequencing to investigate the *MSTN* editing effects on gut microbiota composition and function of sheep, and found that *MSTN* inactivation improved the abundance of beneficial gut microbiota in sheep. The findings in this study provide new insights into the relationship between *MSTN* inactivation and the changes of gut microbiota in sheep.

## Results

### Genotype identification

**Fig. 1:**
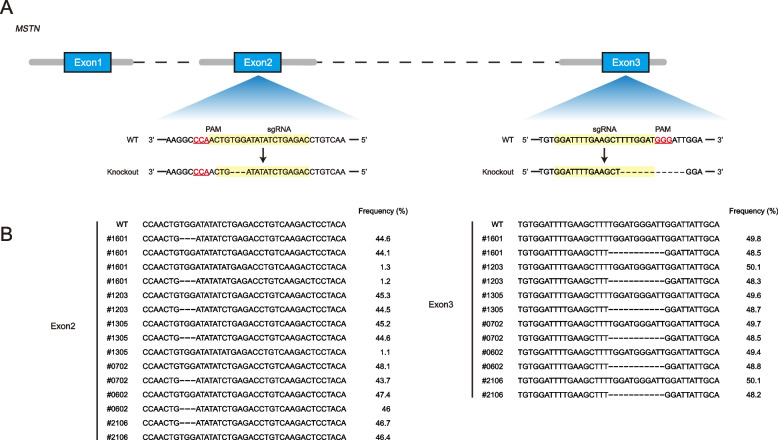
**A** MSTN edited sites in the second and third exons. **B** The sequences of the second and third exons of the MSTN based on deep sequencing

We sequenced the second and third exons of *MSTN*-edited female sheep (GEF), *MSTN*-edited male sheep (GEM), wild-type female sheep (WTF) and wild-type male sheep (WTM). The GEF and GEM groups contained 3 bp (c.469_471delTGG) deletions in the second exon and 11 bp (c.879_889delTGGATGGGATT) deletions in the third exon, but the WTF and WTM groups harbored complete base sequence (Fig. 1; Supplementary Fig. 1).

### Slaughter parameters and meat quality analysis

Three *MSTN*-edited male sheep and three wild-type male sheep were slaughtered and slaughter parameters and mutton quality were tested. The results showed that the body weight (*P* = 0.016), carcass weight (*P* = 0.005) and eye muscle area (*P* = 0.003) of *MSTN*-edited sheep were significantly higher than wild-type sheep (Table 1). However, there were no significant differences in the meat quality indexes (Table 2).

**Table 1:** Effect of *MSTN* editing on slaughter parameters of gene-edited and wild-type sheep

Item	Groups		*P* value
	GEM	WTM	
Body weight (kg)	70.51 ± 3.25	60.15 ± 1.73	0.016
Carcass weight (kg)	39.25 ± 1.04	30.02 ± 2.15	0.005
Dressing percentage (%)	55.60 ± 3.00	50.00 ± 2.00	0.112
Eye muscle area (cm^2^)	29.04 ± 1.12	20.52 ± 1.51	0.003

**Table 2:** Effect of *MSTN* editing on meat quality as assessed on the longissimus dorsi of gene-edited and wild-type sheep

Item	Groups		*P* value
	GEM	WTM	
a*_45 min_^a^	10.16 ± 0.52	10.08 ± 1.41	0.148
b*_45 min_^b^	6.37 ± 0.87	5.86 ± 0.83	0.318
L*_45 min_^c^	29.78 ± 1.75	29.18 ± 0.54	0.417
a* _24 h_^a^	16.91 ± 3.56	15.98 ± 1.21	0.087
b* _24 h_^b^	13.55 ± 1.58	13.14 ± 1.11	0.235
L* _24 h_^3)^	34.37 ± 0.62	36.41 ± 0.90	0.688
pH_45 min_	6.58 ± 0.10	6.44 ± 0.20	0.34
pH_24 h_	6.01 ± 0.24	5.75 ± 0.09	0.15
Water loss rate (%)	4.15 ± 0.87	2.30 ± 0.57	0.53
Cooking loss (%)	48.81 ± 1.13	50.12 ± 0.84	0.065
Intramuscular fat (g/100 g)	13.73 ± 1.62	12.94 ± 4.44	0.509

^a^ Measure of redness (larger number indicates a more intense red color)

^b^ Measure of yellowness (larger number indicates more yellow color)

^c^ Measure of lightness (larger number indicates a lighter color)

### Alpha and beta diversity of the gene-edited and wild-type sheep

**Fig. 2:**
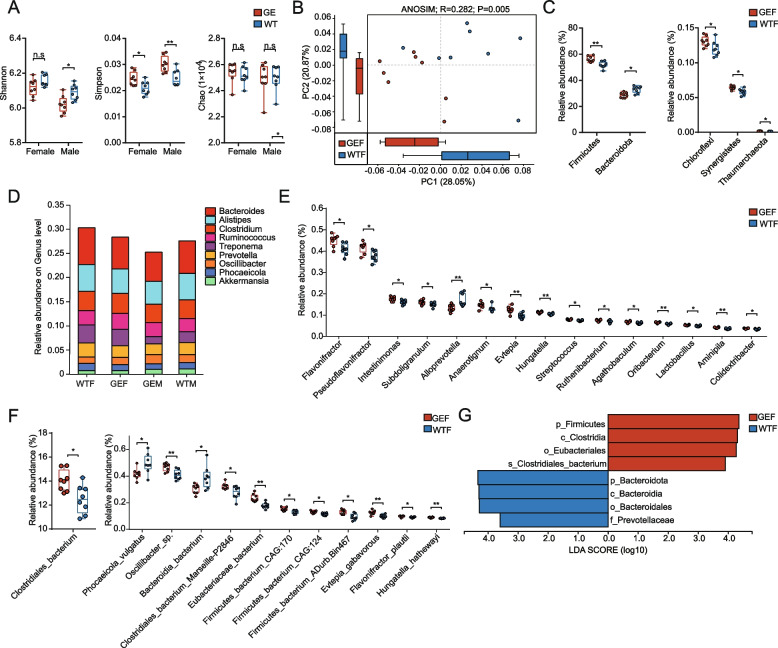
The composition of gut microbiota in the MSTN-edited and wild-type groups. **A** The statistical graphs of alpha diversity of gut microbiota among the GEF, WTF, GEM and WTM. **B** Bray–Curtis-based PCoA of the gut microbiota in the GEF and WTF. The *P*-value was based on ANOSIM. The boxplot shows the discrete distribution of samples along the PC1 and PC2 axes. **C** The significantly different phyla between the GEF and WTF. **D** The histogram of the dominant genera in the GEF, WTF, GEM and WTM. **E** The significantly different genera between the GEF and WTF. **F** The significantly different species between the GEF and WTF. (0.01 < *P* ≤ 0.05 *, 0.001 < *P* ≤ 0.01 **). **G** LEfSe analysis of the GEF and WTF. The histogram shows the microbes that can best illustrate the difference between the GEF and WTF. The larger the LDA score, the greater the contribution of the corresponding microbe to the difference

The gut microbiota of the four groups (GEF, WTF, GEM and WTM) were analysed. Compared to the WTF, the Simpson index of the GEF was significantly higher (*P* = 0.039), and the Chao and Shannon index showed no significant difference (*P* = 0.383, *P* = 0.148). Also, compared to the WTM, the Simpson index of the GEM was significantly higher (*P* = 0.008), the Shannon index was significantly lower (*P* = 0.016), and the Chao index showed no significant difference (*P* = 0.999) (Fig. 2A). Principal coordinates analysis (PCoA) revealed the GEF and WTF were distinct. Analysis of Similarities (ANOSIM) indicated the differences between the GEF and WTF were greater than that within their own (Fig. 2B, R = 0.283, *P* = 0.005). Comparable findings were observed between the GEM and WTM (Supplementary Fig. 2A, R = 0.254, *P* = 0.011).

### Composition of gut microbiota of gene-edited and wild-type sheep

We compared the difference of the abundance of gut microbiota between the GEF and WTF, as well as the GEM and WTM. We observed the *MSTN* editing did not change the dominant phyla of sheep, the top 7 phyla were Firmicutes, Bacteroidota, Spirochaetes, Verrucomicrobia, Proteobacteria, Euryarchaeota and Lentisphaerae (Supplementary Fig. 2B). However, the Wilcoxon rank-sum test revealed that compared to the WTF, the abundance of Firmicutes (*P* = 0.005 in female sheep, *P* = 0.041 in male sheep), Chloroflexi (*P* = 0.04 in female sheep, *P* = 0.007 in male sheep), Synergistetes (*P* = 0.014 in female sheep, *P* = 0.01 in male sheep) and Thaumarchaeota (*P* = 0.01 in female sheep, *P* = 0.007 in male sheep) was significantly higher but that of Bacteroidota (*P* = 0.01 in female sheep, *P* = 0.041 in male sheep) was significantly lower in the GEF (Fig. 2C). The comparison between the GEM and WTM revealed similar findings (Supplementary Fig. 2C). Further analyses revealed that Firmicutes/Bacteroidota (F/B) ratio was significantly higher in the GEF (*P* = 0.016), but there was no statistical difference in the F/B ratio between the GEM and WTM groups (*P* = 0.054). At the genus level, the top 9 dominant genera across the 4 groups were *Bacteroides, Alistipes, Clostridium, Ruminococcus, Prevotella, Treponema, Phocaeicola, Oscillibacter* and *Akkermansia* (Fig. 2D). Compared to the WTF, the abundance of *Flavonifractor* (*P* = 0.014 in female sheep, *P* = 0.007 in male sheep), *Pseudoflavonifractor* (*P* = 0.014 in female sheep, *P* = 0.004 in male sheep), *Intestinimonas* (*P* = 0.031 in female sheep, *P* = 0.003 in male sheep), *Subdoligranulum* (*P* = 0.031 in female sheep, *P* = 0.01 in male sheep), *Anaerotignum* (*P* = 0.041 in female sheep, *P* = 0.031 in male sheep), *Evtepia* (*P* = 0.007 in female sheep, *P* = 0.014 in male sheep), *Hungatella* (*P* = 0.004 in female sheep, *P* = 0.041 in male sheep), *Streptococcus* (*P* = 0.01 in female sheep, *P* = 0.004 in male sheep), *Ruthenibacterium* (*P* = 0.041 in female sheep, *P* = 0.007 in male sheep), *Agathobaculum* (*P* = 0.018 in female sheep, *P* = 0.024 in male sheep), *Oribacterium* (*P* = 0.004 in female sheep, *P* = 0.014 in male sheep), *Lactobacillus* (*P* = 0.018 in female sheep, *P* = 0.01 in male sheep), *Aminipila* (*P* = 0.004 in female sheep, *P* = 0.005 in male sheep) and *Colidextribacter* (*P* = 0.018 in female sheep, *P* = 0.005 in male sheep) was significantly higher, whereas that of *Alloprevotella* (*P* = 0.007 in female sheep, *P* = 0.031 in male sheep) was significantly lower in the GEF (Fig. 2E). Comparable results were observed between the GEM and WTM (Supplementary Fig. 2D). At the species level, the top 10 most abundant species across the four groups included *Clostridiales_*bacterium*, Ruminococcaceae_*bacterium*, Bacteroidales_*bacterium*, Lachnospiraceae_*bacterium*, Clostridia_*bacterium*, Clostridiaceae_*bacterium*, Rikenellaceae_*bacterium*, Firmicutes_bacterium_*CAG:110*, Alistipes_sp._*Z76 and *Treponema_porcinum* (Supplementary Fig. 2E). The abundance of *Clostridiales_*bacterium (*P* = 0.031 in female sheep, *P* = 0.014 in male sheep), *Oscillibacter_*sp*.* (*P* = 0.007 in female sheep, *P* = 0.041 in male sheep), *Clostridiales_bacterium_*Marseille-P2846 (*P* = 0.024 in female sheep, *P* = 0.031 in male sheep), *Eubacteriaceae_*bacterium (*P* = 0.003 in female sheep, *P* = 0.01 in male sheep), *Firmicutes_bacterium_*CAG:170 (*P* = 0.014 in female sheep, *P* = 0.004 in male sheep), *Firmicutes_bacterium_*Adur. Bin467 (*P* = 0.01 in female sheep, *P* = 0.005 in male sheep), *Firmicutes_bacterium_*CAG:124 (*P* = 0.018 in female sheep, *P* = 0.003 in male sheep), *Evtepia_gabavorous* (*P* = 0.007 in female sheep, *P* = 0.014 in male sheep), *Flavonifractor_plautii* (*P* = 0.024 in female sheep, *P* = 0.003 in male sheep) and *Hungatella_hathewayi* (*P* = 0.007 in female sheep, *P* = 0.024 in male sheep) were higher both in the GEF and GEM groups, whereas those of *Phocaeicola_vulgatus* (*P* = 0.031 in female sheep, *P* = 0.041 in male sheep) and *Bacteroidia_*bacterium (*P* = 0.024 in female sheep, *P* = 0.041 in male sheep) were lower both in the GEF and GEM groups (Fig. 2F; Supplementary Fig. 2F).

The LEfSe at LDA > 3 revealed that 8 bacterial taxa including Firmicutes, *Clostridia, Eubacteriales* and *Clostirdiales_*bacterium were significantly more abundant in the GEF, whereas Bacteroidota, *Bacteroidia, Bacteroidales* and *Prevotellaceae* were more abundant in the WTF (Fig. 2G). A comparison of the GEM and WTM revealed comparable findings (Supplementary Fig. 2G).

**Fig. 3:**
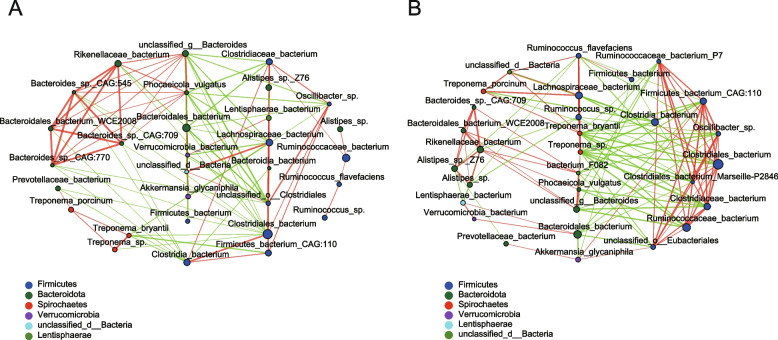
The microbial correlation networks of the (**A**) MSTN-edited groups (including GEF and GEM) and (**B**) wild-type groups (including WTF and WTM). The nodes colored differently represent different phyla; the size of nodes corresponds with the species abundance, the thickness of lines corresponds with the strength of association, the number of lines on the nodes corresponds with the relatedness of the species, the red color represents positive correlations whereas the green color represents negative correlations

In addition, we considered the top 30 species of the four groups for correlation network analysis. In general, *MSTN* mutation had no significant effect on the interaction of gut microbes (Fig. 3).

### MSTN mutation altered potential function of hindgut microbiota based on metagenomic sequencing

**Fig. 4:**
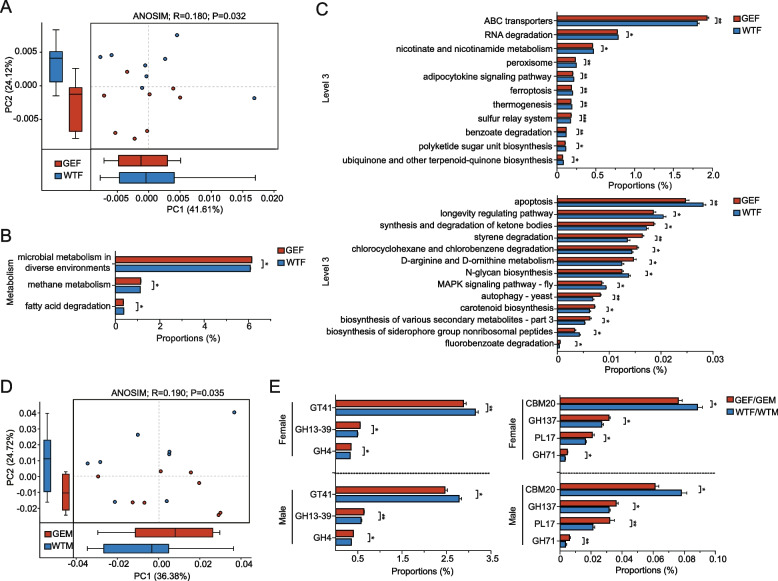
Upregulated and downregulated signaling pathways and CAZymes in the MSTN-edited and wild-type groups. **A** Bray–Curtis-based PCoA of the functional pathways of the GEF and WTF. The *P*-value was based on ANOSIM. The boxplot for the discrete distribution of samples along the PC1 and PC2 axes. **B** Significantly changed pathways of the GEF and WTF at level 1 (metabolism pathways) and (**C**) level 3. The vertical axis represents different pathways, and the horizontal axis represents the proportions of corresponding pathways. (0.01 < *P* ≤ 0.05 *, 0.001 < *P* ≤ 0.01 **, *P* ≤ 0.001 ***). **D** Bray–Curtis-based PCoA of CAZymes of the GEF and WTF. The *P*-value is based on the ANOSIM. The boxplot shows the discrete distribution of samples along the PC1 and PC2 axes. **E** The significantly different CAZymes of gut microbiome between the GEF/GEM and WTF/WTM. The vertical axis represents different CAZymes, and the horizontal axis represents the proportions of corresponding enzymes. (0.01 < *P* ≤ 0.05 *, 0.001 < *P* ≤ 0.01 **)

Given that *MSTN* mutation influences the abundance of the gut microbiota, we performed KEGG analysis to investigate whether the mutation had an effect on the function of the related biological pathways. PcoA revealed a clear distinction between the GEF and WTF. ANOSIM analysis showed that the differences between the two groups were greater than that within their own (Fig. 4A, R = 0.180, *P* = 0.032). The comparison between the GEM and WTM revealed similar results (Supplementary Fig. 3A, R = 0.199, *P* = 0.026). Besides, we found a significant raising of methane metabolism (*P* = 0.024 in female sheep, *P* = 0.024 in male sheep) and a falling of fatty acid degradation (*P* = 0.014 in female sheep, *P* = 0.041 in male sheep) in GEF and GEM (Fig. 4-B, Supplementary Fig. 3-B). At level 3, the top 5 dominant pathways of the 4 groups were metabolic pathways, biosynthesis of secondary metabolites, microbial metabolism in diverse environments, biosynthesis of amino acids and carbon metabolism, indicating that the *MSTN* mutation did not influence the dominance of main pathways. Besides, we dug a total of 24 significantly affected pathways, 11 of them were upregulated in the GEF and GEM, including ABC transporters (*P* = 0.005 in female sheep, *P* = 0.041 in male sheep), sulfur relay system (*P* = 0.001 in female sheep, *P* = 0.01 in male sheep), benzoate degradation (*P* = 0.005 in female sheep, *P* = 0.01 in male sheep), synthesis and degradation of ketone bodies (*P* = 0.024 in female sheep, *P* = 0.01 in male sheep), styrene degradation (*P* = 0.003 in female sheep, *P* = 0.014 in male sheep), chlorocyclohexane and chlorobenzene degradation (*P* = 0.024 in female sheep, *P* = 0.002 in male sheep), D-arginine and D-ornithine metabolism (*P* = 0.01 in female sheep, *P* = 0.018 in male sheep), autophagy-yeast (*P* = 0.003 in female sheep, *P* = 0.01 in male sheep), carotenoid biosynthesis (*P* = 0.014 in female sheep, *P* = 0.018 in male sheep), biosynthesis of various secondary metabolites-part3 (*P* = 0.031 in female sheep, *P* = 0.004 in male sheep) and fluorobenzoate degradation (*P* = 0.031 in female sheep, *P* = 0.041 in male sheep). Another 13 pathways including RNA degradation (*P* = 0.031 in female sheep, *P* = 0.004 in male sheep), nicotinate and nicotinamide metabolism (*P* = 0.018 in female sheep, *P* = 0.014 in male sheep), peroxisome (*P* = 0.004 in female sheep, *P* = 0.031 in male sheep), adipocytokine signaling pathway (*P* = 0.003 in female sheep, *P* = 0.031 in male sheep), ferroptosis (*P* = 0.007 in female sheep, *P* = 0.031 in male sheep), thermogenesis (*P* = 0.005 in female sheep, *P* = 0.005 in male sheep), polyketide sugar unit biosynthesis (*P* = 0.041 in female sheep, *P* = 0.041 in male sheep), ubiquinone and terpenoid-quinone biosynthesis (*P* = 0.018 in female sheep, *P* = 0.018 in male sheep), apoptosis (*P* = 0.003 in female sheep, *P* = 0.018 in male sheep), longevity regulating pathway (*P* = 0.018 in female sheep, *P* = 0.005 in male sheep), N-glycan biosynthesis (*P* = 0.018 in female sheep, *P* = 0.018 in male sheep), MAPK signaling pathway–fly (*P* = 0.014 in female sheep, *P* = 0.024 in male sheep) and biosynthesis of siderophore group nonribosomal peptides (*P* = 0.024 in female sheep, *P* = 0.041 in male sheep) were downregulated in the GEF and GEM (Fig. 4C, Supplementary Fig. 3C).

### MSTN mutation altered the metabolic activities of CAZymes

Changes of the abundance of CAZymes can indirectly reflect the alterations of gut microbiota. According to PcoA, we observed a clear distinction of CAZymes between the GEF and WTF. ANOSIM analysis revealed that the differences between these two groups were greater than that within their own (Fig. 4D, R = 0.190, *P* = 0.035). Similar results were certified in the GEM and WTM. (Supplementary Fig. 3D, R = 0.197, *P* = 0.034). We found a total of 7 significant changed enzymes, and the abundance of the GH13_39 (*P* = 0.01 in female sheep, *P* = 0.007 in male sheep), GH4 (*P* = 0.014 in female sheep, *P* = 0.041 in male sheep), GH137 (*P* = 0.041 in female sheep, *P* = 0.041 in male sheep), GH71 (*P* = 0.018 in female sheep, *P* = 0.007 in male sheep) and PL17 (*P* = 0.018 in female sheep, *P* = 0.004 in male sheep) were upregulated, the GT41 (*P* = 0.004 in female sheep, *P* = 0.031 in male sheep) and CBM20 (*P* = 0.024 in female sheep, *P* = 0.01 in male sheep) were downregulated (Fig. 4E).

## Discussion

Whether *MSTN*-edited sheep will degrade their meat quality and taste is an important question. In this study, we analyzed the meat color, pH, water loss rate and cooking loss and proved that there was no difference in meat quality indices between the *MSTN*-edited sheep and the wild-type sheep.

Gender affects the composition of microbes, and sex hormones are one of the important reasons for this difference. A study analyzed the 16S rRNA dataset of the human microbiome project (HMP) and found that male stool tended to have a higher abundance of *Prevotella* and *Bacteroides* [22]. Another study with a larger individual showed significantly different of gut microbiome composition of female and male, and female group harbored higher α diversity [23]. In animal studies, α diversity of cecal microbiota in 10-week-old female mice was significantly higher than that in 10-week-old male mice. PCA analysis showed that female and castrated male microbiota were closer to each other than to male microbiota [24]. To eliminate the potential influence of gender on the results, we sequenced the gut microbiome of *MSTN*-edited male sheep, wild-type male sheep, *MSTN*-edited female sheep and wild-type female sheep, and selected the microbes with the same change trend in different genders for further analysis. The results revealed that *MSTN* inactivation had no significant effect on the key dominant gut microbiota in sheep, consistent with previous research in pigs [20, 25]. However, PcoA analysis revealed a clear distinction between the *MSTN*-edited and wild-type sheep, suggesting that the gut microbiome readily adjusts its structure and physiological functions to suit the physiological changes caused by the genetic alteration in the host [26, 27].

Specifically, in phylum level, the abundance of Firmicutes increased, whereas that of Bacteroidota decreased in the *MSTN*-edited sheep. The F/B ratio, in the *MSTN*-edited sheep, was higher, which have been reported in the obese humans and animals relative to their counterparts with normal weight. This suggests a higher F/B ratio is a biomarker for excessive body fats (obesity and overweight) [28–30]. In contrast, however, conflicting findings have been reported. For instance, some researchers found no difference of F/B ratio in the lean and obsess individuals [31, 32]. In addition, lower F/B ratio in obese animals and humans have also been reported [33]. Following slaughter it was determined that the body weight, carcass weight and the eye muscle area increased significantly in the *MSTN*-edited sheep relative to the wild-type sheep. Given that Firmicutes is efficient energy-harvesting bacteria [29, 34], we hypothesized the higher abundance of Firmicutes in the *MSTN*-edited sheep is an adaptation to meet the increased energy requirements for bigger muscle gain. Bacteroidota have been reported to promote inflammation, and Bacteroidota are more abundant in low-gene-count (LGC) individuals than in high-gene-count (HGC) individuals [35]. This suggests fewer Bacteroidota is a biomarker for a healthy gut.

In genus level, the abundance of some microbes including *Flavonifractor, Subdoligranulum, Ruthenibacterium, Agathobaculum, Anaerotignum, Oribacterium* and *Lactobacillus*, was significantly higher in the *MSTN*-edited sheep than those in the wild-type sheep. These microbes play a crucial role for their hosts in the synthesis of acetate, propionate and butyrate [36–44]. These short-chain fatty acids (SCFAs) are not only secreted and absorbed by the gut as nutrients, but they are also important in maintaining the health of the hosts. For example, butyrate is the key regulator for the homeostasis, endocrine and immune function [45, 46]. Specifically, it lowers the risk of colon cancer [47–51], modulates inflammation related to bacterial infection and participates in maintaining the body mass index (BMI) [35]. In addition, we found *MSTN* mutation increased the abundance of Thaumarchaeota, an archaebacterium that participates in the glycolysis, oxidative phosphorylation, vitamin production and nitrification [52, 53].

Furthermore, KEGG analysis revealed the downregulation of lipid metabolism, inflammation and apoptosis signaling pathways and upregulation of vitamin synthesis pathway, which are the positive signal for the gut health of the *MSTN*-edited sheep. But these findings need to be validated with further metabolomics data.

## Conclusions

In conclusion, our results showed that *MSTN* inactivation remarkably influenced the composition and potential function of hindgut microbial communities of the sheep and significantly promoted growth performance without affecting meat quality. Our results demonstrate that the different microbial composition in the hindgut may provide positive signal for the hindgut health and nutrition absorption for the host of *MSTN*-edited sheep. The findings of this study provide new insights into the relationship between *MSTN* mutation and the gut microbiota in sheep and preliminarily prove the quality of *MSTN*-edited mutton has no different from that of wild type mutton.

## Materials and methods

### The experimental animals and sample collection

In this study, *MSTN* gene-edited sheep were used as experimental group, and wild-type sheep were used as control group. Considering the possible influence of gender and age as well as other factors on the results, we selected 8 *MSTN*-edited female sheep (GEF), 8 wild-type female sheep (WTF), 8 *MSTN*-edited male sheep (GEM) and 8 wild-type male sheep (WTM). All of these sheep were 1.5 years old, lived at the Ningxia Tianyuan Sheep Farm (Wuzhong, Ningxia, China) and housed in pens (6 m × 9 m; *n* = 4 per pen) under controlled environmental conditions. They were provided with enough food and water. The feeds were formulated meeting or exceeding the current feeding recommendations (Supplementary Table 1). The blood samples for DNA extraction and genotyping were extracted from the jugular veins into anticoagulant vacutainer tubes. The slaughtered sheep were from the GEM and WTM. The mutton samples for quality analysis were obtained from *longissimus dorsi* of slaughtered sheep. The fecal samples for metagenomic sequencing were collected from the rectum of the experimental sheep and stored in sterile 10 mL tubes in liquid nitrogen pending extraction of microbial DNA.

### Genotype identification

These experimental sheep were the offspring of *MSTN*^±^ male sheep carrying 3 bp (c.469_471delTGG) deletions in the second exon and 11 bp (c.879_889delTGGATGGGATT) deletions in the third exon of the *MSTN* and wild-type females. In order to ensure the accuracy of the sheep grouping, we verified the genotypes by sequencing. Total DNA was extracted using the Blood Genomic DNA Mini Kit (CoWin Biosciences, Shanghai, China). The *MSTN* gene was amplified using the 2 × Rapid Taq Master MIX (Vazyme Biotech Co., Ltd., Nanjing, China). The PCR products were separated and assessed on 1.0% agarose gel. Sanger sequencing was performed at Sangon Biotech Co., Ltd. (Shanghai, China). Deep sequencing was performed at Novogene Co., Ltd. (Tianjin, China).

### Determination of slaughter parameters and meat quality

The experimental sheep were slaughtered on the same day by following the Halal slaughtering procedure, which involved exsanguination without electrical stunning. After skinning and removing heads, hooves and internal organs, the carcasses were weighted and the slaughter rate (carcass weight/body weight) were calculated. Finally, *longissimus dorsi* muscles were isolated for meat color, pH, water loss rate, cooking loss, intramuscular fat and eye muscle area measuring. Meat quality analysis was performed as previously described [54]. Briefly, the meat color (L*, a* and b*) and pH values at 45 min and 24 h in *longissimus dorsi* were measured by using a Minolta CM-600D spectrophotometer (Konica Minolta Sensing Inc., Osaka, Japan) and a pH–STAT meter (SFK-Technology, Denmark), respectively. For the water loss rate, a circular sampler was used to cut the *longissimus dorsi* about 20 g and an analytical balance MS1602S (Thermo Fisher Scientific, Waltham, MA, USA) was used to weight the pre-pressure weight. Then the sample was sandwiched into the multi-layer filter papers, pressed 35 kg with water loss rate tester Bulader-M10 (Bulader, Beijing, China) for 5 min and reweighted the sample. Water loss rate was expressed as a percentage of weight change before and after pressure. For the cooking loss analysis, 100 g of muscle was weighed and cooked in a thermostat water bath DK-S22 (Shanghai Jing Hong Laboratory Instrument Co., Ltd., Shanghai, China) at 85 ℃ for 50 min. After that, the sample was removed, blotted dry on filter paper, and reweighted. Cooking loss was expressed as a percentage of weight change before and after cooking. In addition, intramuscular fat content was determined by the soxhlet extraction method. *Longissimus dorsi* were lyophilized to a constant weight (no evaporable moisture remaining) using vacuum freeze dryer (SCIENTZ-18ND, Ningbo, Zhejiang, China), and then subjected to ether extraction using the Soxhlet apparatus and diethyl ether. After 8 h of extraction, the samples were removed, air-dried and reweighed to determine fat loss. Moreover, the eye muscle area was measured by pasting a sulfate paper on the cross section of the muscle and measuring the area of the outline of the muscle. The data are expressed as the mean ± SEM and analyzed using Student’s t-test with significant differences considered at *P* < 0.05.

### Metagenomic sequencing, assembly, and construction of the gene library

To construct the paired-end (PE) library, DNA extracted from the GEF, WTF, GEM and WTM groups were fragmented into small 300 bp segments using the Covaris M220 machine (Gene, Hong Kong, China). The PE library was then constructed using the TruSeq DNA Sample Prep Kit (Illumina, San Diego, CA, USA), following the manufacturer’s instructions. Sequencing of the PE reads was performed using the Illumina HiSeq 4000 platform (Illumina, San Diego, CA, USA). A total of 2,269,248, 212 raw reads were obtained. The minimum number of raw reads per group was 61,035,344 but averaged 70,914,007. Adapter sequences were removed from the 3’ and 5’ ends of the raw reads. Low-quality raw reads (length < 50 bp, quality values < 20, or containing N bases) were also removed using the FastP software (Version 0.20.0) [55]. A total of 2,235,699,610 clean reads were retained (98.52% of raw reads). The minimum number of clean reads in a given sample was 60,093,748 but averaged 69,865,613. After removing sequences for the *Ovis aries* genome (assembly ARS-UI_Ramb_v2.0) sequences with BWA (version 0.7.9a) [56]. A total of 1,841,359,674 high quality reads, representing 81.17% of total raw reads, were obtained. A minimum of 40,225,450, reads, but an averagely of 57,542,490 per sample, were cleaned. Contigs were generated using the Megahit software (Version 1.1.2) [57]. Only 25,233,472 contigs larger than 300 bp were retained. The minimum number of contigs per sample was 561,569 but averaged 788,546. The average size of the contigs was 208,436 bp. The maximum N50 and N90 were 705 bp and 357 bp, the minimum N50, and N90 were 547 bp and 338 bp, and the average N50 and N90 were 622 bp and 347 bp. The open reading frames (ORFs) in contigs were predicted using the MetaGene software [58]. A total of 57,239,602 ORFs larger than 100 bp, which were translated into amino acid sequences at the National Center for Biotechnology Information (NCBI), were retained. The maximum ORFs ranged between 100 bp and 27,414 bp but averaged 440 bp. All predicted genes with a 90% sequence identity (90% coverage) were clustered into groups using the CD-HIT software (version 4.6.1) [59]. The final non-redundant gene sets contained 25,836,001 ORFs, which was 45.14% of the original ORFs. The average length of ORFs was 497 bp. To evaluate gene abundances in each sample, quality ORFs within each sample were mapped to the corresponding sequence with 95% identity using SOAP aligner (Version 2.21) [60]. For taxonomic annotations, selected non-redundant gene sets were blasted in the NCBI-NR database using the DIAMOND software (parameter: BLASTP; E-value ≤ 1e^−5^) [61, 62]. A total of 5 domains, 13 kingdoms, 235 phyla, 449 classes, 895 orders, 1,779 families, 5,369 genera and 35,877 species were annotated. Similarly, for KEGG pathway analysis [63], non-redundant gene sets were blasted in the GENES using the DIAMOND software (parameter: BLASTP; E-value ≤ 1e^−5^), and for CAZymes, non-redundant gene sets were blasted in the CAZy database (E-value ≤ 1e^−5^) using the HMMSCAN [64].

### Statistical analysis

The difference in gene expression between the GEF and WTF groups, GEM and WTM groups were analyzed using the Wilcoxon rank-sum test at two-tailed *P*-values. The gene expression data were presented using box-and-whisker plots. Alpha and beta diversity indices (Bray–Curtis) were calculated using the Mothur software (Version 1.30.2) and R (Version 3.3.1). The differences in the bacterial taxa between different groups were assessed using Analysis of similarity (ANOSIM) of Bray–Curtis distances. Data analysis and FDR correction of the *p*-values were performed using the R package (Version 3.3.3). The operational taxonomic units most likely to explain differences between classes by coupling standard tests for statistical significance with additional tests encoding biological consistency and effect relevance were identified based on Linear discriminant analysis (LDA) effect size (LEfSe). The correlation network analysis was performed using the Networks software (Version 2.1).

## Supplementary Information

**Additional file 1:** **Supplementary Fig. 1.** The sequences of the second and third exons of MSTN based on Sanger sequencing.

**Additional file 2:** **Supplementary Fig. 2.** The composition of gut microbiota in the MSTN-edited and wild type-groups. A. Bray-Curtis based PCoA of the gut microbiome in the GEM and WTM. The *P*-value was based on ANOSIM. The boxplot shows the discrete distribution of samples along the PC1 and PC2 axes. B. The histogram of the dominant phyla in the GEF, WTF, GEM and WTM. It excludes unclassified microbes. C. The significantly different phyla between the GEM and WTM. D. The significantly different genera between the GEM and WTM. E. The histogram of the dominant species in the GEF, WTF, GEM and WTM. It excludes unclassified microbes. F. The significantly different species between the GEM and WTM. G. LEfSe analysis of the GEM and WTM. The histogram shows the microbes that can best illustrate the difference between the GEM and WTM. The larger the LDA score, the greater the contribution of the corresponding microbe to the difference.

**Additional file 3:** **Supplementary Fig. 3.** Upregulated and downregulated signaling pathways and CAZymes in the GEM and WTM. A. Bray-Curtis-based PCoA of the functional pathways. The *P*-value was based on ANOSIM. The boxplot for the discrete distribution of samples along the PC1 and PC2 axes. B. Significantly changed pathways at level 1 (metabolism pathways) and (C) level 3. The vertical axis represents different pathways, and the horizontal axis represents the proportions of corresponding pathways. (0.01< *P* ≤0.05 *, 0.001< *P* ≤0.01 **, *P* ≤0.001 ***). D. Bray-Curtis-based PCoA of CAZymes. The *P*-value is based on the ANOSIM. The boxplot shows the discrete distribution of samples along the PC1 and PC2 axes. 

**Additional file 4:** **Supplementary Table 1.** Composition and nutrient levels of basal diet. 

## Data Availability

The datasets analysed during the current study are available in the national center for biotechnology information (NCBI) under accessions number PRJNA783381 (https://www.ncbi.nlm.nih.gov/bioproject/PRJNA783381).
