# Supplementary material for: Inactivation of the MSTN gene expression changes the composition and function of the gut microbiome in sheep
Source: BMC Microbiol. 2022 Nov 11;22:273. doi: 10.1186/s12866-022-02687-8 (PMC9650872; doi:10.1186/s12866-022-02687-8)
Supplement: Supplementary file 1 — Additional file 1: Supplementary Fig. 1. The sequences of the second and third exons of MSTN based on Sanger sequencing. [file 12866_2022_2687_MOESM1_ESM.pdf]

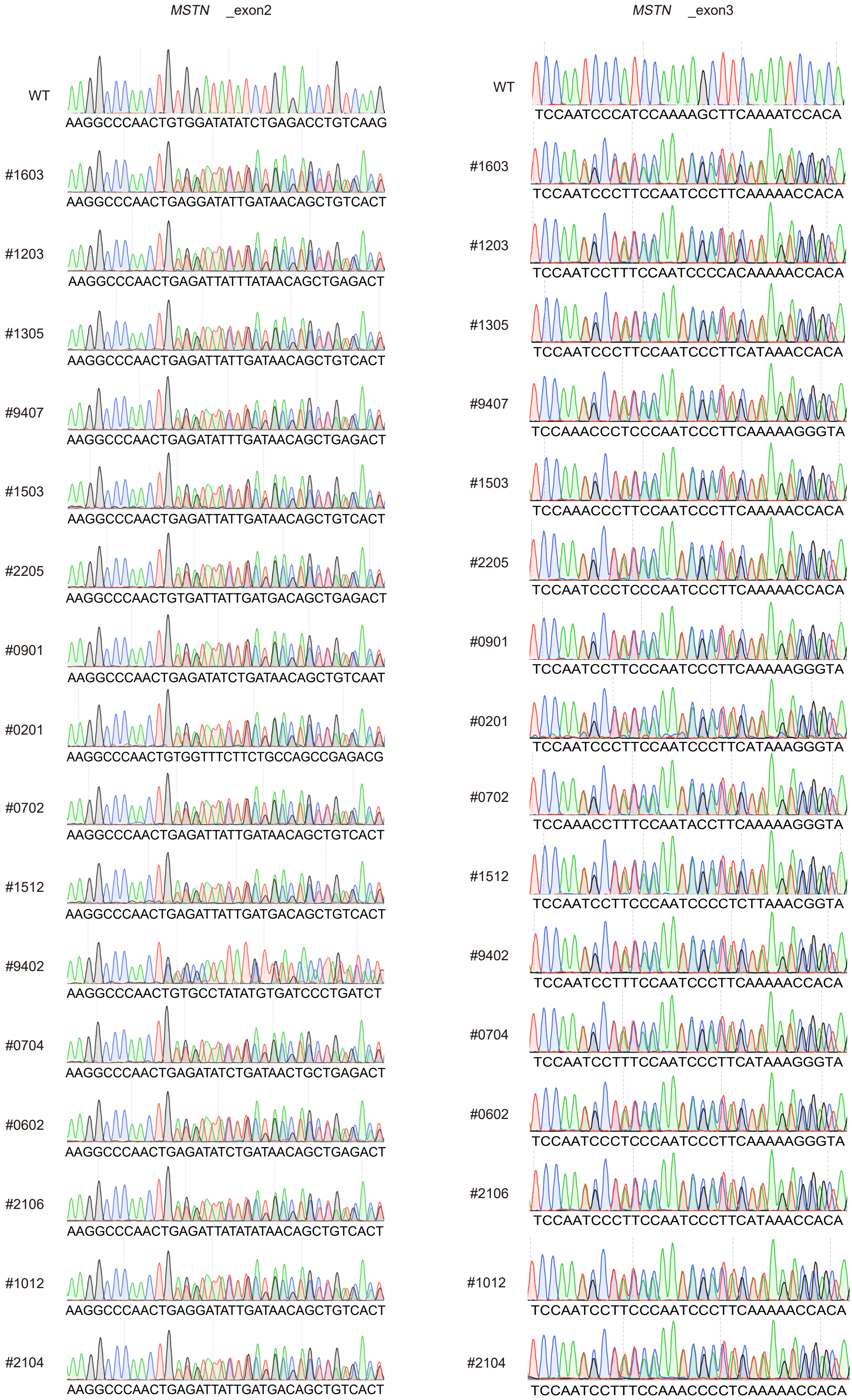

Supplementary Fig. 1. The sequences of the second and third exons of MSTN based on Sanger sequencing.
