# Supplementary material for: Inactivation of the MSTN gene expression changes the composition and function of the gut microbiome in sheep
Source: BMC Microbiol. 2022 Nov 11;22:273. doi: 10.1186/s12866-022-02687-8 (PMC9650872; doi:10.1186/s12866-022-02687-8)
Supplement: Supplementary file 2 — Additional file 2: Supplementary Fig. 2. The composition of gut microbiota in the MSTN-edited and wild type-groups. A. Bray-Curtis based PCoA of the gut microbiome in the GEM and WTM. The P-value was based on ANOSIM. The boxplot shows the discrete distribution of samples along the PC1 and PC2 axes. B. The histogram of the dominant phyla in the GEF, WTF, GEM and WTM. It excludes unclassified microbes. C. The significantly different phyla between the GEM and WTM. D. The significantly different genera between the GEM and WTM. E. The histogram of the dominant species in the GEF, WTF, GEM and WTM. It excludes unclassified microbes. F. The significantly different species between the GEM and WTM. G. LEfSe analysis of the GEM and WTM. The histogram shows the microbes that can best illustrate the difference between the GEM and WTM. The larger the LDA score, the greater the contribution of the corresponding microbe to the difference. [file 12866_2022_2687_MOESM2_ESM.pdf]

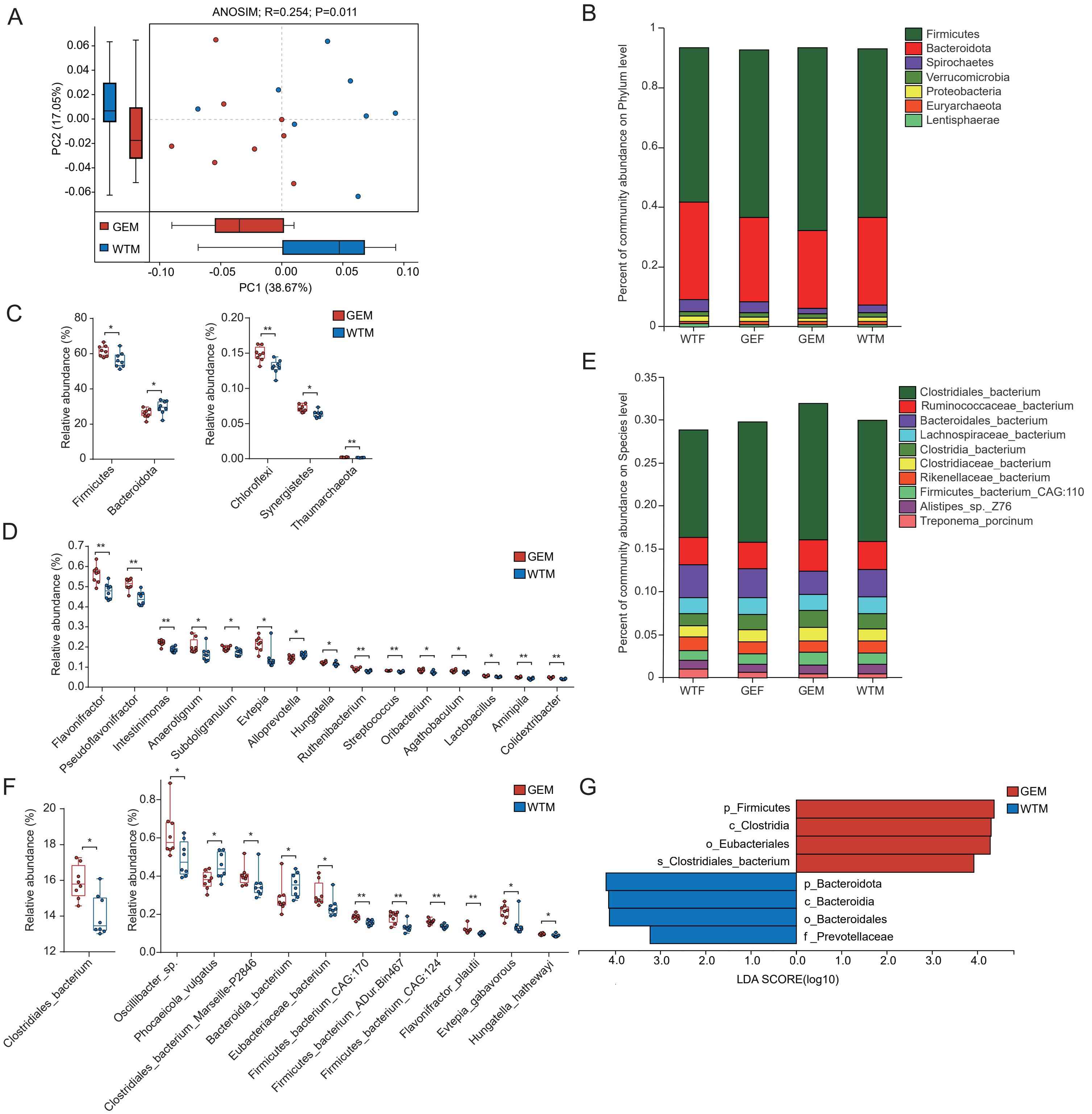

Supplementary Fig. 2 The composition of gut microbiota in the MSTN-edited and wild type-groups. A. Bray-Curtis-based PCoA of the gut microbiome in the GEM and WTM. The P-value was based on ANOSIM. The boxplot shows the discrete distribution of samples along the PC1 and PC2 axes. B. The histogram of the dominant phyla in the GEF, WTF, GEM and WTM. It excludes unclassified microbes. C. The significantly different phyla between the GEM and WTM. D. The significantly different genera between the GEM and WTM. E. The histogram of the dominant species in the GEF, WTF, GEM and WTM. It excludes unclassified microbes. F. The significantly different species between the GEM and WTM. G. LEfSe analysis of the GEM and WTM. The histogram shows the microbes that can best illustrate the difference between the GEM and WTM. The larger the LDA score, the greater the contribution of the corresponding microbe to the difference.
