# Supplementary material for: Inactivation of the MSTN gene expression changes the composition and function of the gut microbiome in sheep
Source: BMC Microbiol. 2022 Nov 11;22:273. doi: 10.1186/s12866-022-02687-8 (PMC9650872; doi:10.1186/s12866-022-02687-8)
Supplement: Supplementary file 3 — Additional file 3: Supplementary Fig. 3. Upregulated and downregulated signaling pathways and CAZymes in the GEM and WTM. A. Bray-Curtis-based PCoA of the functional pathways. The P-value was based on ANOSIM. The boxplot for the discrete distribution of samples along the PC1 and PC2 axes. B. Significantly changed pathways at level 1 (metabolism pathways) and (C) level 3. The vertical axis represents different pathways, and the horizontal axis represents the proportions of corresponding pathways. (0.01< P ≤0.05 *, 0.001< P ≤0.01 **, P ≤0.001 ***). D. Bray-Curtis-based PCoA of CAZymes. The P-value is based on the ANOSIM. The boxplot shows the discrete distribution of samples along the PC1 and PC2 axes. [file 12866_2022_2687_MOESM3_ESM.pdf]

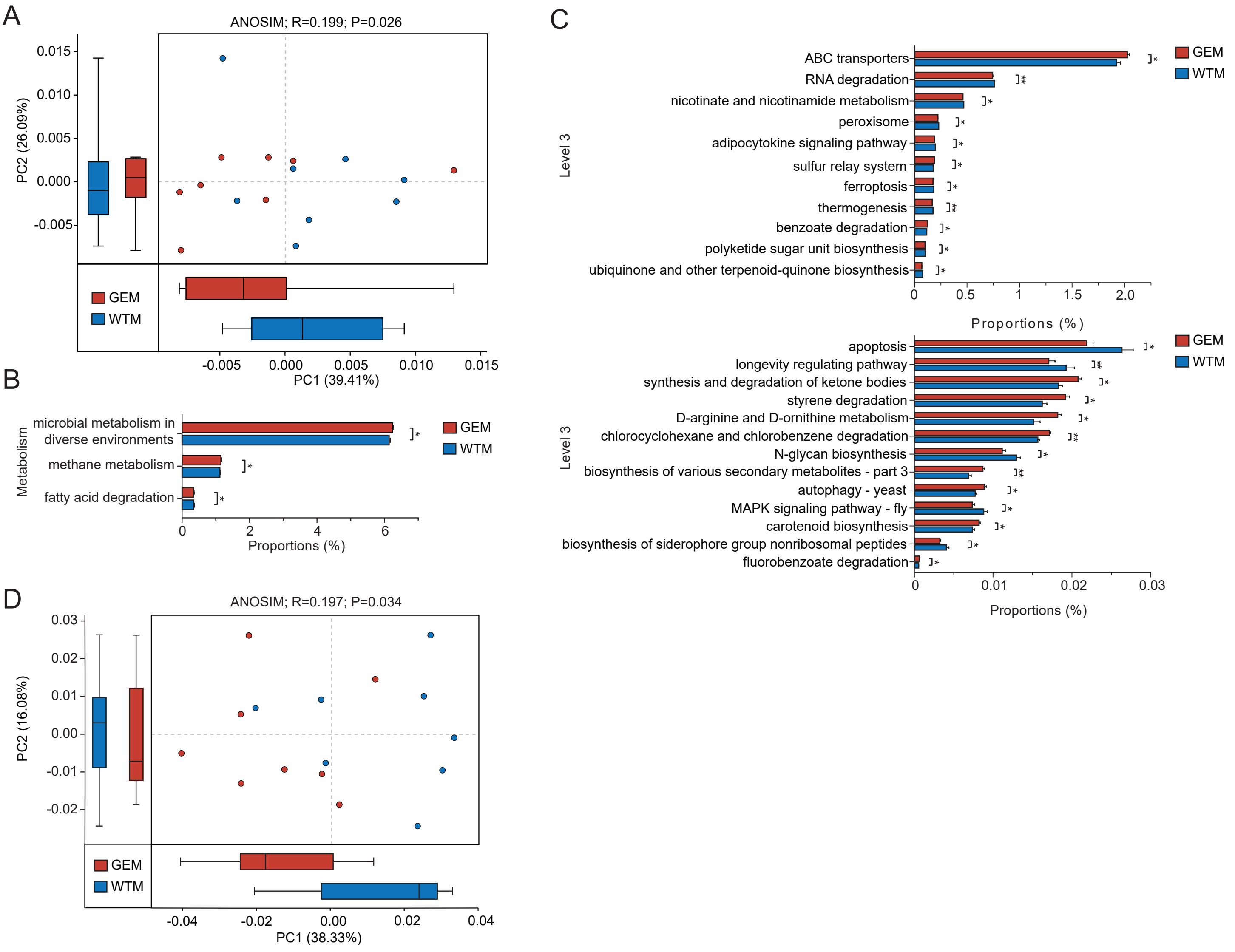

Supplementary Fig. 3 Upregulated and downregulated signaling pathways and CAZymes in the GEM and WTM. A. Bray-Curtis-based PCoA of the functional pathways. The P-value was based on ANOSIM. The boxplot for the discrete distribution of samples along the PC1 and PC2 axes. B. Significantly changed pathways at level 1 (metabolism pathways) and (C) level 3. The vertical axis represents different pathways, and the horizontal axis represents the proportions of corresponding pathways. (0.01< P ≤0.05 *, 0.001< P ≤0.01 **, P ≤0.001 ***). D. Bray-Curtis-based PCoA of CAZymes. The P-value is based on the ANOSIM. The boxplot shows the discrete distribution of samples along the PC1 and PC2 axes.
