# Supplementary material for: Inactivation of the MSTN gene expression changes the composition and function of the gut microbiome in sheep
Source: BMC Microbiol. 2022 Nov 11;22:273. doi: 10.1186/s12866-022-02687-8 (PMC9650872; doi:10.1186/s12866-022-02687-8)
Supplement: Supplementary file 4 — Additional file 4: Supplementary Table 1. Composition and nutrient levels of basal diet. [file 12866_2022_2687_MOESM4_ESM.docx]

Supplementary Table 1 Composition and nutrient levels of basal diet

| Item | % |
| --- | --- |
| Ingredients |  |
| Corn grain | 35.00 |
| Soybean meal | 14.46 |
| Alfalfa meal | 20.00 |
| Corn stalk | 27.54 |
| CaHPO4 | 1.00 |
| Premix (5%)^1)^ | 2.00 |
| Total | 100.00 |
| Nutrient levels |  |
| DM^2)^ | 83.10 |
| DE/(MJ/kg)^3)^ | 10.38 |
| CP^4)^ | 14.36 |
| EE^5)^ | 2.38 |
| NDF^6)^ | 31.09 |
| ADF^7)^ | 16.32 |
| Ca | 0.75 |
| TP^8)^ | 0.52 |

^1)^One kilogram premix included: FeSO_4_·7H_2_O 2%, CuSO_4_·5H_2_O 0.5%, MnSO_4_·5H_2_O 1%, ZnSO_4_·7H_2_O 1%, KI 0.005%, Na_2_SO_3_·5H_2_O 0.004%, V_A_ 0.1%, V_D3_ 0.02%, V_E_ 0.4%, Monensin sodium 0.2%, Carrier 92.97%. DM, CP were measured, DE were calculated.

^2)^DM, Dry matter

^3)^DE, Digestible Energy

^4)^CP, Crude Protein

^5)^EE, Ether Extract

^6)^NDF, Neutral Detergent Fiber

^7)^ADF, Acid Detergent Fiber

^8)^TP, Total Phosphorous

**Supplementary Table 1.** The composition and levels of nutrients of the basal feeds.
